# Supplementary figures and images for: Salivary microbiome profiles for different clinical phenotypes of pituitary adenomas by single-molecular long-read sequencing
Source: Microbiol Spectr. 2023 Oct 6;11(6):e00234-23. doi: 10.1128/spectrum.00234-23 (PMC10715156; doi:10.1128/spectrum.00234-23)

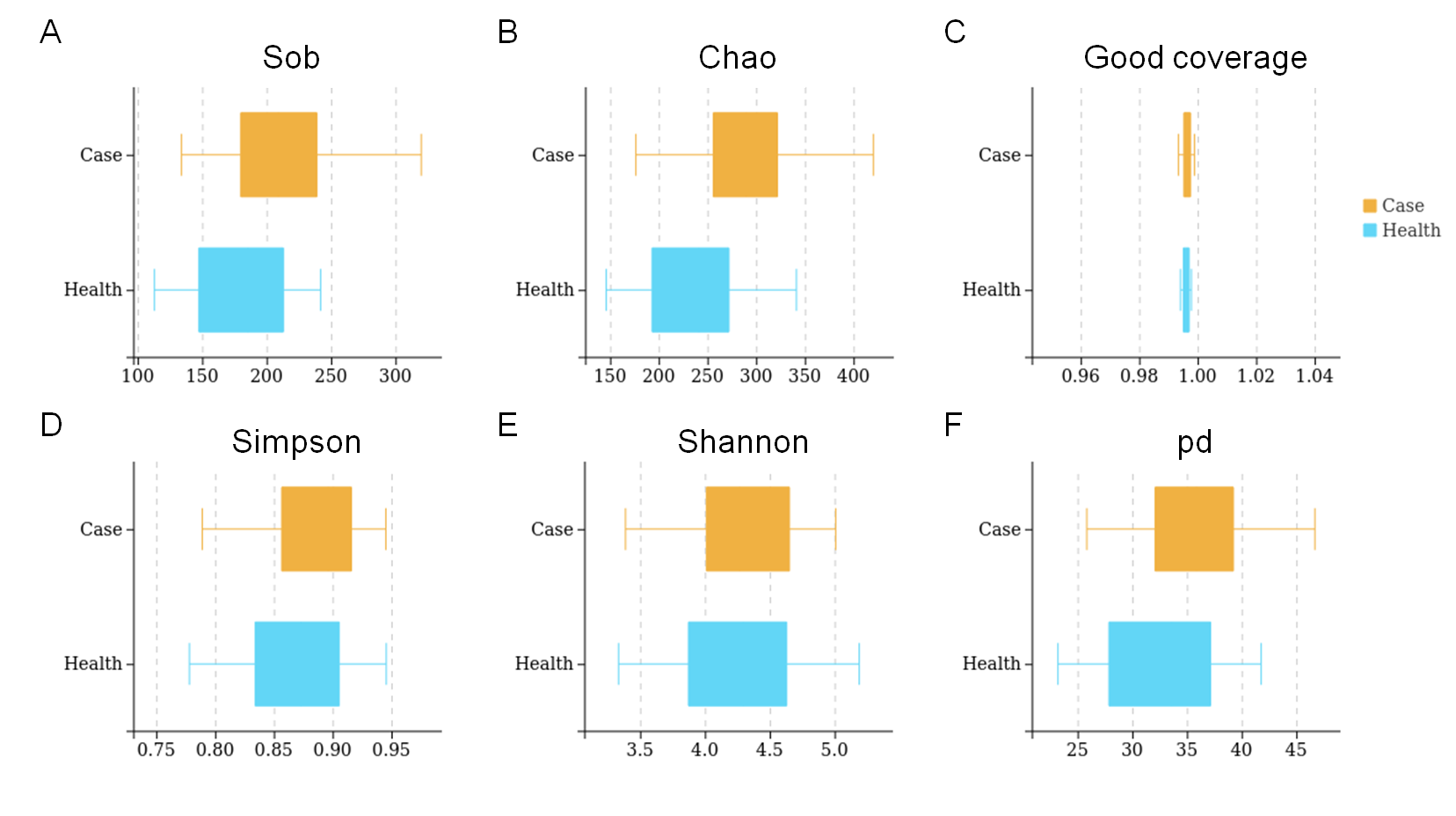

Supplement: Supplemental Figure S1 — Supplementary Figure S1. α-Diversity analysis of the observed species (A), Chao1 index (B), Good's coverage (C), Simpson index (D), Shannon index (E), and PD-whole tree index (F) between the groups of PA patients and healthy individuals. [file spectrum.00234-23-s0001.tif]

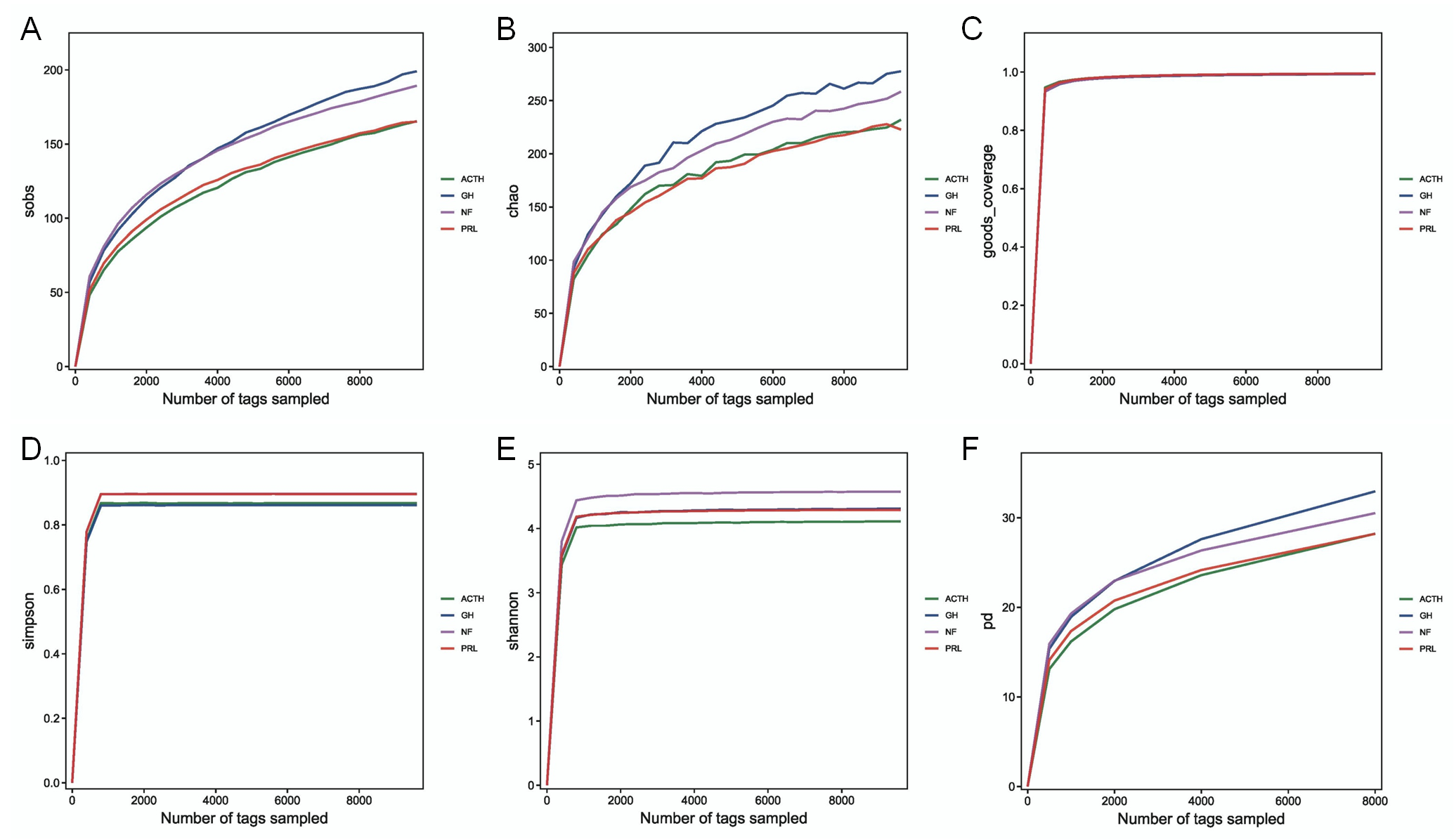

Supplement: Supplemental Figure S2 — α-Diversity analysis of the observed species (A), Chao1 index (B), Good's coverage (C), Simpson index (D), Shannon index (E), and PD-whole tree index (F) for the different groups of samples. [file spectrum.00234-23-s0002.tif]

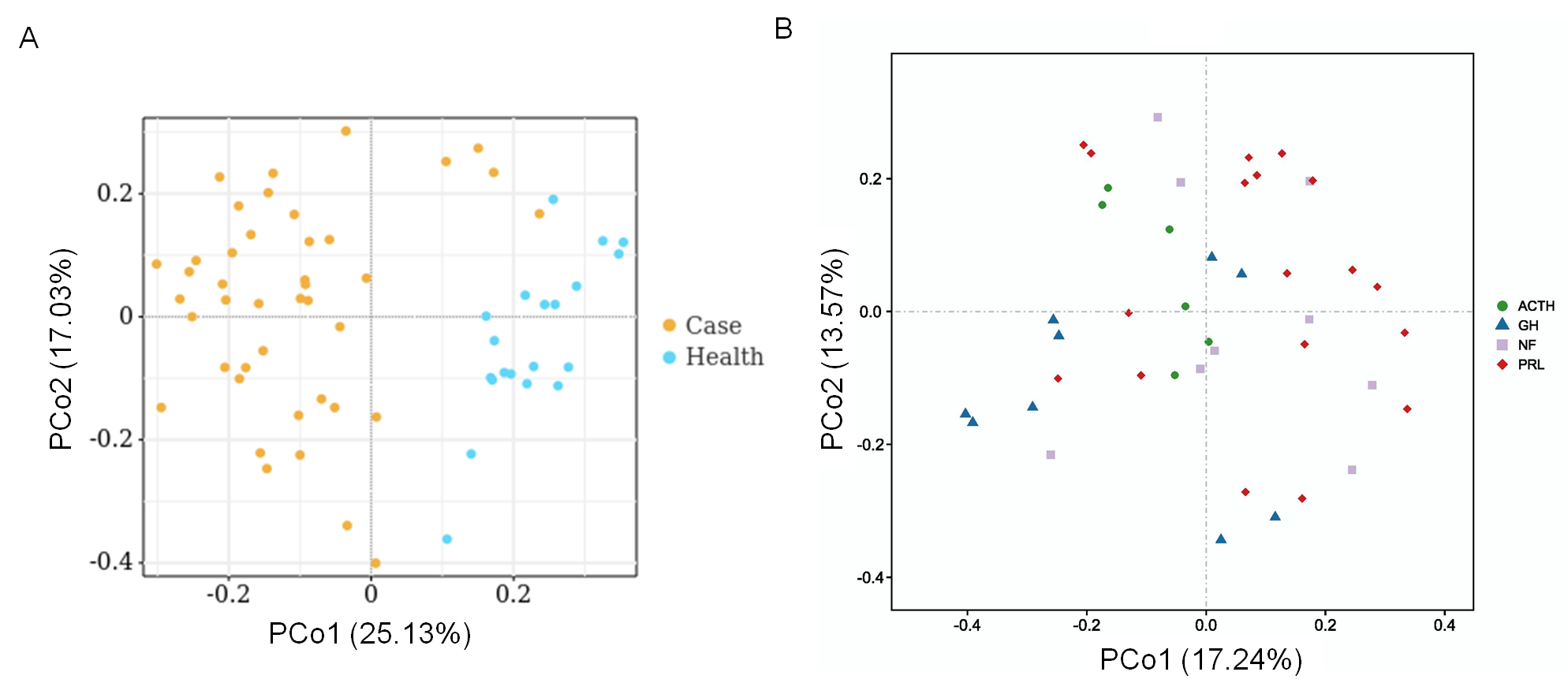

Supplement: Supplemental Figure S3 — β-Diversity analysis based on principal coordinate analysis (PCoA) of the samples. (A) PCoA between the groups of PA patients and healthy individuals. (B) PCoA among the four groups of PA patients. [file spectrum.00234-23-s0003.tif]

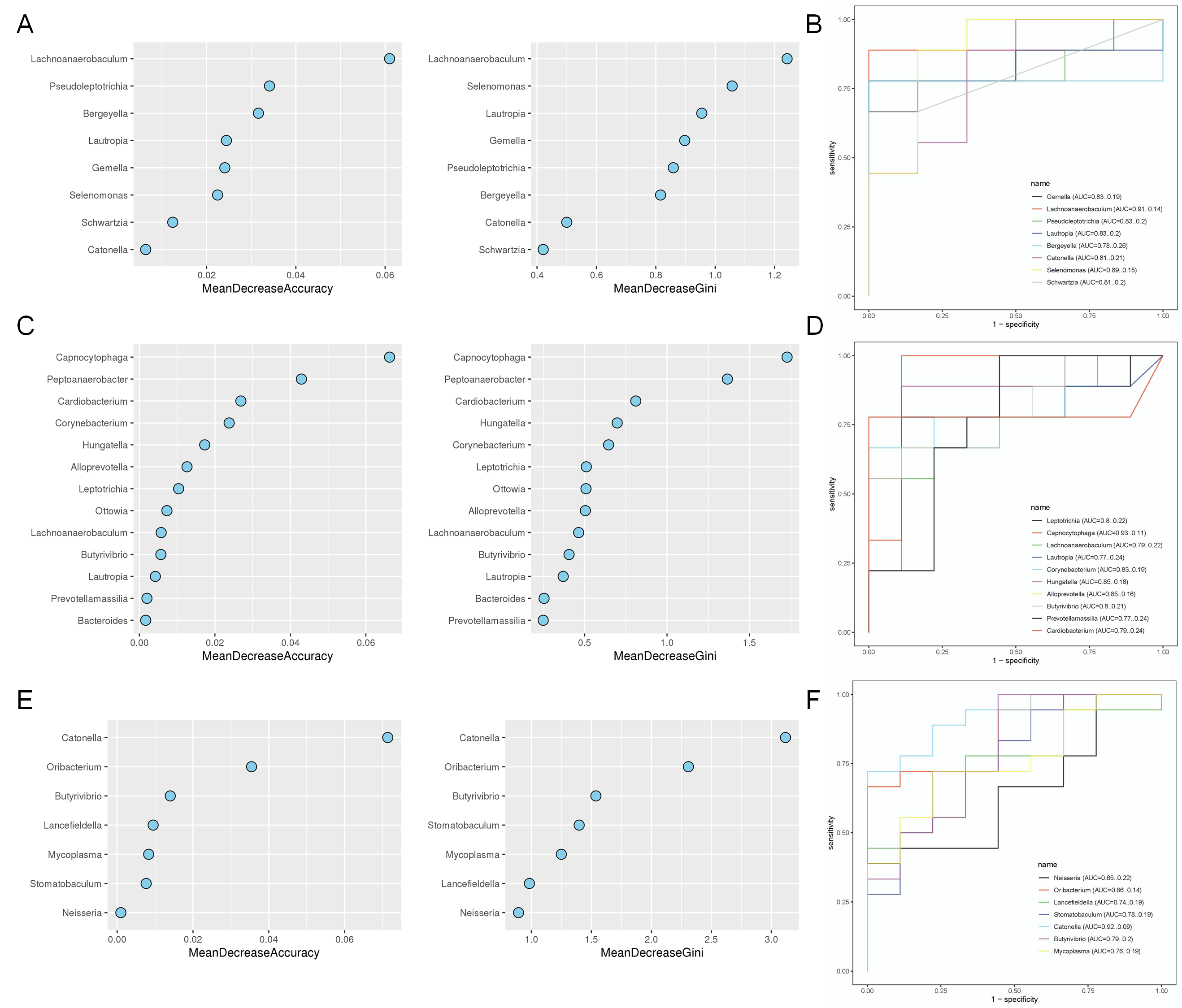

Supplement: Supplemental Figure S4 — Indicator salivary microbes corresponding to the genus phylotype among the different groups. (A) Random-forest analysis of the biomarkers between ACTH-PA and NF-PA. (B) Receiver operating curve (ROC) analysis of the biomarkers between ACTH-PA and NF-PA. (C) Random-forest analysis of the biomarkers between GH-PA and NF-PA. (D) ROC analysis of the biomarkers between GH-PA and NF-PA. (E) Random-forest analysis of the biomarkers between PRL-PA and NF-PA. (F) ROC analysis of the biomarkers between PRL-PA and NF-PA. [file spectrum.00234-23-s0004.tif]

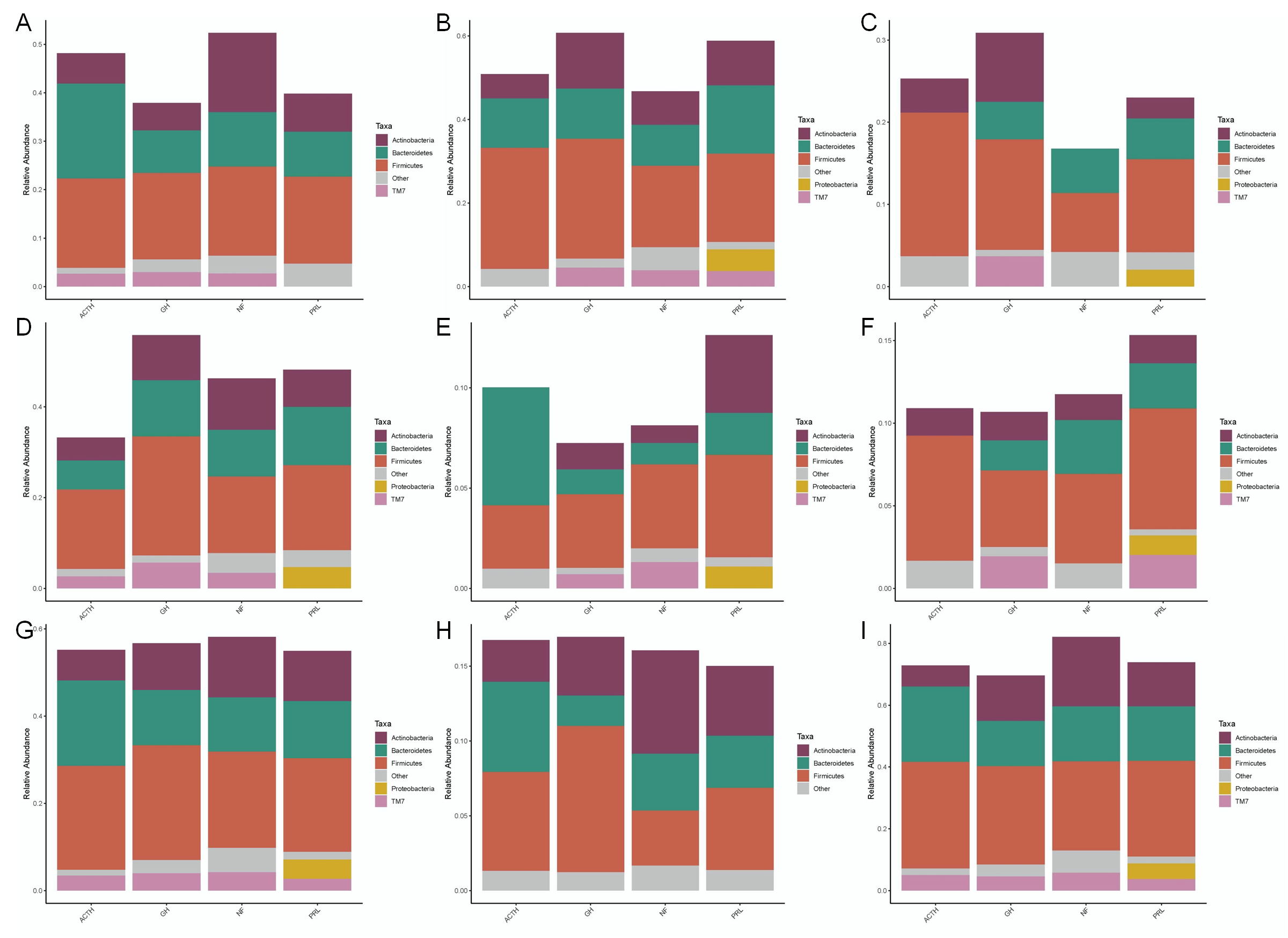

Supplement: Supplemental Figure S5 — Supplemental Figure 5. Phenotype prediction for the microbial species among the different groups of PA. (A) Gram-positive; (B) Gram-negative; (C) biofilm-forming; (D) pathogenic; (E) mobile element-containing; (F) aerobic; (G) anaerobic; (H) facultatively anaerobic; (I) oxidative stress-tolerant. [file spectrum.00234-23-s0005.tif]
