# Supplementary material for: Salivary microbiome profiles for different clinical phenotypes of pituitary adenomas by single-molecular long-read sequencing
Source: Microbiol Spectr. 2023 Oct 6;11(6):e00234-23. doi: 10.1128/spectrum.00234-23 (PMC10715156; doi:10.1128/spectrum.00234-23)
Supplement: Supplemental legends — Legends of supplemental figures and table. [file spectrum.00234-23-s0006.docx]

**Legends**

**Supplemental Figure S1.** α-Diversity analysis of the observed species (A), Chao1 index (B), Good’s coverage (C), Simpson index (D), Shannon index (E), and PD-whole tree index (F) between the groups of PA patients and healthy individuals.

**Supplemental Figure S2.** α-Diversity analysis of the observed species (A), Chao1 index (B), Good’s coverage (C), Simpson index (D), Shannon index (E), and PD-whole tree index (F) for the different groups of samples

**Supplemental Figure S3.** β-diversity analysis based on principal coordinate analysis (PCoA) of the samples. A: PCoA analysis between the groups of PA patients and healthy individuals. B: PCoA analysis among the four groups of PA patients.

**Supplemental Figure S4.** Indicator salivary microbes corresponding to the genus phylotype among the different groups. A: Random-forest analysis of the biomarkers between ACTH-PA and NF-PA. B: Receiver operating curve (ROC) analysis of the biomarkers between ACTH-PA and NF-PA. C: Random-forest analysis of the biomarkers between GH-PA and NF-PA. D: ROC analysis of the biomarkers between GH-PA and NF-PA. E: Random-forest analysis of the biomarkers between PRL-PA and NF-PA. F: ROC analysis of the biomarkers between PRL-PA and NF-PA.

**Supplemental Figure S5.** Phenotype prediction for the microbial species among the different groups of PA. A: Gram positive; B: Gram negative; C: biofilm-forming; D: pathogenic; E: mobile element-containing; F: aerobic; G: anaerobic; H: facultatively anaerobic; I: oxidative stress-tolerant.

**Supplemental Table S1:** The list of common and unique microbes among different groups of patients with pituitary adenoma.
