# Supplementary material for: Salivary microbiome profiles for different clinical phenotypes of pituitary adenomas by single-molecular long-read sequencing
Source: Microbiol Spectr. 2023 Oct 6;11(6):e00234-23. doi: 10.1128/spectrum.00234-23 (PMC10715156; doi:10.1128/spectrum.00234-23)
Supplement: Supplemental table — S1: The list of common and unique microbes among different groups of patients with pituitary adenoma. [file spectrum.00234-23-s0007.docx]

**Supplementary Table 1:** The list of common and unique microbes among different groups of patients with pituitary adenoma.

| **Common bacteria among 4 clinical phenotypes of PA** | |
| --- | --- |
|  | Abiotrophia |
|  | Actinomyces |
|  | Alloprevotella |
|  | Aminipila |
|  | Arachnia |
|  | Bergeyella |
|  | Bulleidia |
|  | Butyrivibrio |
|  | Campylobacter |
|  | Capnocytophaga |
|  | Cardiobacterium |
|  | Catonella |
|  | Centipeda |
|  | Cloacibacterium |
|  | Clostridiales_Family_XIII._Incertae_Sedis_unclassified |
|  | Corynebacterium |
|  | Defluviitalea |
|  | Dialister |
|  | Filifactor |
|  | Fusobacterium |
|  | Gemella |
|  | Granulicatella |
|  | Haemophilus |
|  | Hungatella |
|  | Kingella |
|  | Lachnoanaerobaculum |
|  | Lancefieldella |
|  | Lautropia |
|  | Leptotrichia |
|  | Mogibacterium |
|  | Mycoplasma |
|  | Neisseria |
|  | Oribacterium |
|  | Ottowia |
|  | Paludibacter |
|  | Parvimonas |
|  | Peptoanaerobacter |
|  | Peptococcus |
|  | Peptostreptococcus |
|  | Porphyromonas |
|  | Prevotella |
|  | Prevotellamassilia |
|  | Pseudoleptotrichia |
|  | Rothia |
|  | Scardovia |
|  | Schaalia |
|  | Selenomonas |
|  | Shuttleworthia |
|  | Solobacterium |
|  | Stomatobaculum |
|  | Streptococcus |
|  | Tannerella |
|  | Treponema |
|  | Unclassified |
|  | Veillonella |
| **Unique bacteria in ACTH-PA** | |
|  | Staphylococcus |
| **Unique bacteria in PRL-PA** | |
|  | Eikenella |
|  | Eubacterium |
|  | Fructilactobacillus |
| **Unique bacteria in GH-PA** | |
|  | Bifidobacterium |
|  | Johnsonella |
|  | Pseudoramibacter |
|  | Slackia |
|  | Sneathia |
| **Unique bacteria in NF-PA** | |
|  | Desulfobulbus |
|  | Ligilactobacillus |
|  | Phocaeicola |
